# Meningococcal virulence in zebrafish embryos depends on capsule polysaccharide structure

**DOI:** 10.3389/fcimb.2022.1020201

**Published:** 2022-09-23

**Authors:** Kim Schipper, Lisanne C. Preusting, Nina M. van Sorge, Yvonne Pannekoek, Arie van der Ende

**Affiliations:** Amsterdam University Medical Centers, Location University of Amsterdam, Amsterdam Infection Immunity, Department of Medical Microbiology and Infection Prevention Netherlands Reference Laboratory for Bacterial Meningitis, Location AMC, Amsterdam, Netherlands

**Keywords:** *Neisseria meningitidis*, zebrafish embryo infection, innate immunity, meningococcal polysaccharide capsule, isogenic capsule variants

## Abstract

*Neisseria meningitidis* or the meningococcus, can cause devasting diseases such as sepsis and meningitis. Its polysaccharide capsule, on which serogrouping is based, is the most important virulence factor. Non-encapsulated meningococci only rarely cause disease, due to their sensitivity to the host complement system. How the capsular polysaccharide structure of *N. meningitidis* relates to virulence is largely unknown. Meningococcal virulence can be modeled in zebrafish embryos as the innate immune system of the zebrafish embryo resembles that of mammals and is fully functional two days post-fertilization. In contrast, the adaptive immune system does not develop before 4 weeks post-fertilization. We generated isogenic meningococcal serogroup variants to study how the chemical composition of the polysaccharide capsule affects *N. meningitidis* virulence in the zebrafish embryo model. H44/76 serogroup B killed zebrafish embryos in a dose-dependent manner, whereas the non-encapsulated variant was completely avirulent. Neutrophil depletion was observed after infection with encapsulated H44/76, but not with its non-encapsulated variant HB-1. The survival of embryos infected with isogenic capsule variants of H44/76 was capsule specific. The amount of neutrophil depletion differed accordingly. Both embryo killing capacity and neutrophil depletion after infection correlated with the number of carbons used per repeat unit of the capsule polysaccharide during its biosynthesis (indicative of metabolic cost).

**Conclusion:**

Meningococcal virulence in the zebrafish embryo largely depends on the presence of the polysaccharide capsule but the extent of the contribution is determined by its structure. The observed differences between the meningococcal isogenic capsule variants in zebrafish embryo virulence may depend on differences in metabolic cost.

## Introduction

*Neisseria meningitidis*, often referred to as the meningococcus, is an obligatory human pathogen, which can cause severe invasive meningococcal diseases (IMD) such as meningitis and septicemia (van de Beek et al., 2021). Based on the structure of the capsular polysaccharide (CPS), 13 serogroups are defined, of which 6 (serogroup A, B, C, X, Y and W) are causing most of the disease. Except for serogroup B, conjugated polysaccharide vaccines have been developed and implemented in immunization programs (Pizza et al., 2020).

The serogroup B, C, W and Y capsules all contain sialic acid [N-acetylneuraminic acid (Neu5Ac)] in a specific configuration. The serogroup B meningococcal polysaccharide capsule is a homopolymer of α-2-8-linked Neu5Ac and is not O-acetylated (Bhattacharjee et al., 1975). It is poorly immunogenic, as it is identical to polysialic acid structures expressed on human neuronal cells (Finne et al., 1983). Thus, protein-based vaccines against serogroup B meningococci were developed (Pizza et al., 2020). The serogroup C meningococcal polysaccharide capsule is a homopolymer of α-2-9-linked Neu5Ac (Bhattacharjee et al., 1975) and acetylated at position C7 or C8 (Lemercinier and Jones, 1996). The serogroup W and Y polysaccharides are both heteropolymers composed of α-2-6-linked Neu5Ac and galactose or glucose, respectively. The Neu5Ac moieties can be acetylated at position C7 in case of serogroup W polysaccharide and at position 9 for serogroup Y polysaccharide (Bhattacharjee et al., 1976; Lemercinier and Jones, 1996). In part of serogroup C, W and Y isolates the capsule polysaccharide is not O-acetylated, due to phasevariable expression of the O-acetyltranferase or absence of the gene encoding it (Borrow et al., 2000; Longworth et al., 2002).

Genes that are involved in CPS biosynthesis and cell surface translocation are clustered together in the CPS locus. The loci of all 6 invasive serogroups are divided into 6 regions; in order D, A, C, E, D’ and B (Harrison et al., 2013) (**Supplemental Figure 2A**). All regions, except region A, are conserved within each serogroup. Region A differs per serogroup as it contains allgenes involved in the serogroup specific CPS synthesis (Harrison et al., 2013).

The meningococcus has to overcome the host innate immune system to survive and multiply in the blood stream of the host (Lewis and Ram, 2020). This is illustrated by the observation that complement-deficient individuals are more susceptible to IMD (Mikucki et al., 2022). The meningococcal polysaccharide capsule is the most important virulence factor, through its ability to confer serum resistance (Tzeng et al., 2016). Non-encapsulated meningococci are susceptible to complement and do not survive in the bloodstream. A study by Ram et al. showed that capsular polysaccharides differ in their activation of the alternative complement pathway, which, in turn, may contribute to differences between serogroups in pathogenesis (Ram et al., 2011). However, how the chemical composition of the different serogroup-defining capsular polysaccharides affects meningococcal virulence is currently unknown.

The immune system of the zebrafish embryo resembles that of mammals (Stream and Madigan, 2022). During the first several days of zebrafish development, a functional adaptive immune system is absent until 4 weeks post-fertilization, while innate immune cells and complement provide immune protection. Zebrafish have conserved innate immune receptors, including Toll-like receptors, which function similarly to those in humans (Stream and Madigan, 2022). In addition, neutrophils and macrophages resemble their human counterparts (Stream and Madigan, 2022). As a model species, the zebrafish has additional benefits, including offspring number, *in vitro* fertilization and embryo transparency (van der Sar et al., 2004). Due to its transparency and the availability of a wide range of available fluorescent tools, the zebrafish embryo infection model offers the unique ability to study host-pathogen interaction in real time. Here we developed and used the zebrafish embryo as a meningococcal infection model to study host-pathogen interaction. We generated isogenic serogroup C, W and Y capsule variants of serogroup B *N. meningitidis* H44/76, allowing to study the effect of capsule polysaccharide structure on meningococcal virulence in zebrafish embryos independently of the meningococcal genetic background.

## Methods and materials

### Bacterial strains and growth conditions

In this study we used the *N. meningitidis* H44/76 (B: P1.7,16: F3-3: ST-32 (cc32)) wild type strain and its non-encapsulated mutant HB-1 (Bos and Tommassen, 2005). Bacteria were plated on GC plates (Difco) supplemented with 1% Vitox (Oxoid) and grown overnight at 37°C. Red fluorescent strains were obtained by transforming these strains with pGMC5 (mCherry) through natural transformation (Soyer et al., 2014). The fluorescent genes were inserted between the *recC* and *mrtF* genes in the genome. Transformants were selected on GC agar supplemented with 1% Vitox plates with 10 μg/mL chloramphenicol. Integration was confirmed by Sanger sequencing. For zebrafish infection experiments meningococci were grown on GC plates supplemented with 1% Vitox overnight at 37°C in a humidified atmosphere of 5% CO_2_. Single colonies were collected from an overnight culture and suspended to an optical density (O.D. 600 nm) of 0.1 in 25 ml GC+ 1% Vitox. Bacteria were grown for 3 hours to mid log phase and subsequently harvested by centrifugation (10 minutes, 4000xg). Bacterial pellets were washed twice with Phosphate Buffered Saline (PBS), suspended in PBS and shaken for 30 seconds at 3500 rpm in the magnalyser (Roche) to remove clumps and adjusted to the desired concentration (O.D. 600 nm = 0.35; ~1x10^8^ colony forming units (cfu)/ml) in PBS with 0.25% phenol red (pH 7-7.3 in H_2_O, Sigma-Aldrich). The inoculum was serial diluted and plated on GC plates to determine the number of colony-forming units (cfu).

### Generation of isogenic capsule variants of *N. meningitidis* H44/76

We used three different clinical isolates, a serogroup C, serogroup W and serogroup Y (**Supplemental Material Table 1**) as donor to generate capsule locus fusion fragments between each of the aforementioned serogroups and serogroup B obtained from H44/76. The capsule locus fusion fragments were generated in four steps by PCR according to a method earlier described (Shevchuk et al., 2004). A detailed protocol is presented in Supplemental Materials (**Supplemental Tables 1, 2** and **Supplemental Figures 1, 2**). Through natural transformation, each of the capsule fusion fragments was transformed to H44/76 to generate H44/76_W, H44/76_C and H44/76_Y (**Supplemental Material, Table, Figure 2**).

### Zebrafish embryo care

Adult zebrafish were handled in compliance with the local animal welfare regulations approved by the local animal welfare committee (DEC) and were maintained according to standard protocols (www.zfin.org). Experiments with zebrafish embryos younger than 5 days post fertilization (5 dpf) were excepts from ethical approval. Eggs were harvested within 1 hour after they were spawned and kept in E3 medium (www.zfin.org) at 28 ˚C before further use. For survival experiments, wild type TL lines were used. For confocal imaging, a Transgenic (Tg) line with green fluorescent neutrophils (mpo:eGFP) was used (Renshaw et al., 2006).

### Embryo survival experiments

Embryos were mechanically dechorionated prior to injection. At 28 hours post fertilization (hpf), embryos were anesthetized with 0.4% tricaine in H_2_O and injected in the caudal vein with 1 nl of bacterial suspension or mock injected with 0.25% phenol red in PBS. The number of cfu is depicted in each experiment. Injected embryos were kept individually in 24 well plates and were visually inspected every 24 hours up to 96 hours post infection (hpi). Experiments were performed in triplicates using 20 embryos per condition.

### Culturing bacteria from zebrafish embryos

Embryos were injected in the caudal vein 28 hpf with either mCherry expressing H44/76 or mCherry expressing HB-1 and kept in a group of 10 embryos in a 6 well plate with E3 medium at 28 ˚C. At the indicated time points, embryos were anesthetized with tricaine and individually collected in 200 µl E3 medium. Embryos were mechanically disrupted using the MagNa Lyser (Roche) and shaken 2 times at 3500 rpm for 30 seconds. Serial dilutions were prepared and plated on GC plates (Difco) supplemented with 1% Vitox (Oxoid) and incubated overnight at 37 ˚C in a humidified atmosphere of 5% CO_2_. The next day, fluorescent meningococcal colonies were counted under a fluorescent microscope (Leica, M80), to distinguish between the embryo flora and injected meningococci.

### Imaging by fluorescence microscopy

Individual embryos were placed in a drop of E3 medium and analyzed by fluorescence microscopy (Leica, M80).

### Neutrophil quantification

The Tg line with green fluorescent neutrophils (mpo:eGFP) (Renshaw et al., 2006) was injected 28 hpf with red fluorescent H44/76 or HB-1, or mock injected with 0.25% phenol red in PBS. 24 hpi the embryos were imaged under the fluorescent microscope (Leica, LM8). Images were analyzed for the green fluorescence by using ImageJ version 1.50i. Briefly, images were opened in ImageJ, channels were split and the threshold of the green channel was adjusted and set for all images and the integrated density was measured within the area of interest (Abramoff et al., 2004)

### Statistics

Statistics were performed with Prism (Graphpad v9.1.0.221). Log-rank (Mantel-Cox) test was performed on the survival curves (α=0.01). Significance between means of different groups were assessed with ordinary one-way ANOVA (α=0.05).

## Results

### Zebrafish larvae are susceptible to intravenous injection with encapsulated *meningococci*

**Figure 1:**
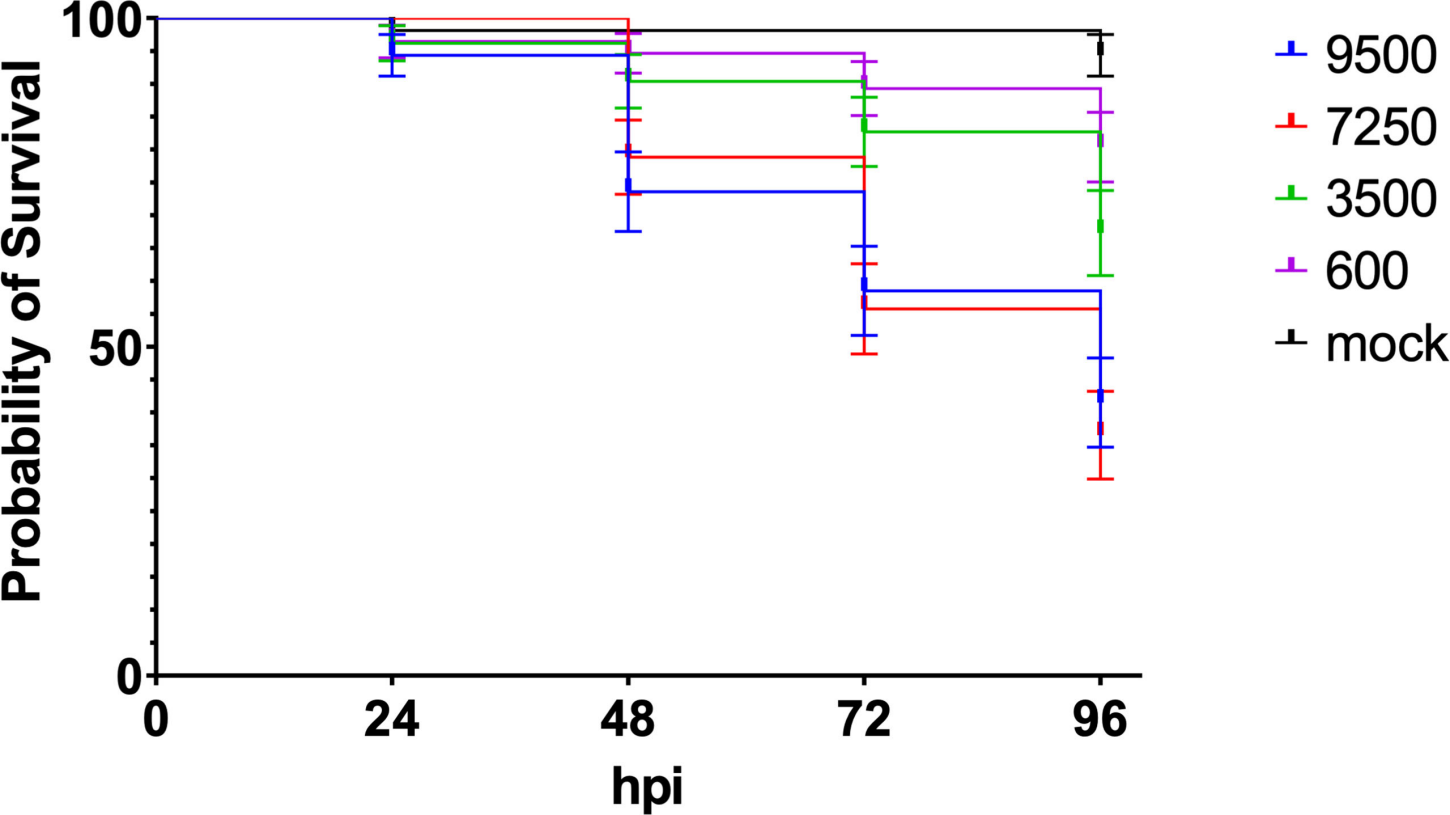
Dose dependent killing of zebrafish embryos by H44/76_B expressing mCherry (numbers in cfu, 20 embryos in each group). Triplicate with 20 embryos in each group.

Zebrafish embryo killing by mCherry expressing meningococci (H44/76 serogroup B; H44/76_B) was dose dependent. Injection with increasing number of cfus in the caudal vein of 28 hpf zebrafish embryos resulted in a dose-dependent infection and mortality of the embryos (**Figure 1** **).** Most of the fish embryos cleared the meningococci when injected with 600 cfu. Fifty percent of the embryos were killed within 96 hpi when infected with an inoculum between 3.5x10^3^ and 7.3x10^3^ cfu.

**Figure 2:**
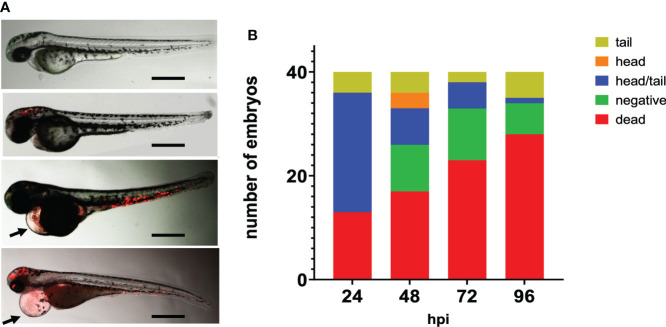
Fluorescence microscopy of zebrafish embryos at 28 hpf infected with mCherry expressing H44/76_B meningococci (5000 cfu). **(A)** Zebrafish embryos infected with red fluorescent meningococci. From top to bottom: No meningococci visible, meningococci in the head, meningococci in the tail and meningococci throughout the body. Images taken at 48 hpi; arrow head shows pericardial edema. Bar represents 750 μm. **(B)** Distribution of meningococci in infected zebrafish embryos.

After 24 hpi multiplication and dissemination of meningococci were visible. After 24 hpi with 5000 cfu, 23% of the fish embryos showed meningococci throughout the body, while in 4% of the embryos, meningococci were only visible in the tail (**Figures 2A, B**). After 48 hpi, meningococci were observed in the head of the embryos only (7.5%), in the tail only (10%) or throughout the body (17.5%) (**Figure 2B**). Part of the embryos (22.5%) had cleared the infection from 48 hpi onwards. Fifty percent of the infected fish embryos showed pericardial edema from 24 hpi onwards (**Figure 2A** **).**

**Figure 3:**
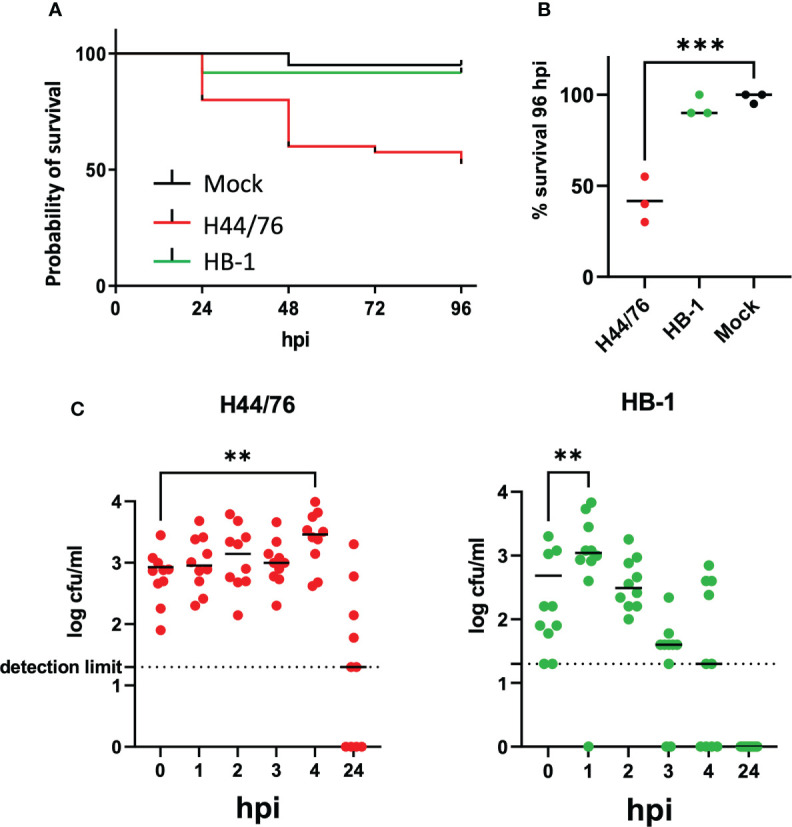
**(A)** Survival curve of zebrafish embryos infected with capsulated H44/76 (9600 cfu) or non-encapsulated meningococci HB-1 (5200 cfu); 20 embryos in each group. **(B)** Survival scored at 96 hpi (right panel). Zebrafish embryos were infected with mCherry-expressing H44/76_B (2700 ± 1820 cfu), HB-1 (1960 ± 1420 cfu) or mock-infected. Three independent experiments each with 20 embryos in each group. **(C)**. Survival and multiplication of meningococci (H44/76_B) or non-encapsulated meningococci (HB-1) in zebrafish embryos. Zebrafish embryos were infected with mCherry expressing H44/76_B (3000 cfu) or HB-1 (2800 cfu); 10 embryos in each group. ** P = < 0.01, ***: P = < 0.001.

Invasive meningococcal disease caused by non-encapsulated meningococci is rare, due to their extreme sensitivity to complement mediated killing. To assess contribution of the meningococcal capsule to meningococcal virulence in the zebrafish embryo infection model, we infected the embryos with 2700 cfu of H44/76_B or with 1960 cfu of the isogenic non-encapsulated variant HB1 and compared the embryo killing capacity. Both variants grow equally well in Tryptic Soy Broth (TSB) medium at 28°C (**Supplemental Figure 3**). The survival at 96 hpi of embryos infected with the wt or the non-encapsulated HB-1 was 51 ± 2%, and 84 ± 5%, respectively, while 93 ± 4% of mock-infected embryos survived (p<0.05) (**Figures 3A, B** **)**. The embryos that survived the infection with HB-1 showed no fluorescent meningococci, indicating that they cleared the infection. In addition, pericardial edema was not observed.

To monitor the multiplication of bacteria in the fish embryo, the number of viable bacteria within embryos at various time points was assessed by plating serial dilutions of homogenates of euthanized embryos onto bacterial culture dishes. Fish embryos were inoculated with 3000 cfu of H44/76 or 2800 cfu of HB-1 to monitor multiplication of the injected meningococci during the first hours post infection. As shown in **Figure 3C** the number of meningococci H44/76_B progressively increased 4-fold in embryos 4 hpi. In contrast, the number of non-encapsulated HB-1 increased in the first hour post infection but then decreased 6-fold at 4 hpi and was 20-fold lower than that of H44/76 (**Figure 3C**; p<0.022). At 24hpi HB-1 could not be recultured from any of the zebrafish embryos, while two of the 10 embryos injected with H44/76_B died and four had countable meningococci. These results are consistent with those presented in **Figure 1**. After infection with 3500 cfu H44/76 or fewer bacteria only a few of the fish embryos had died at 24 hpi.

**Figure 4:**
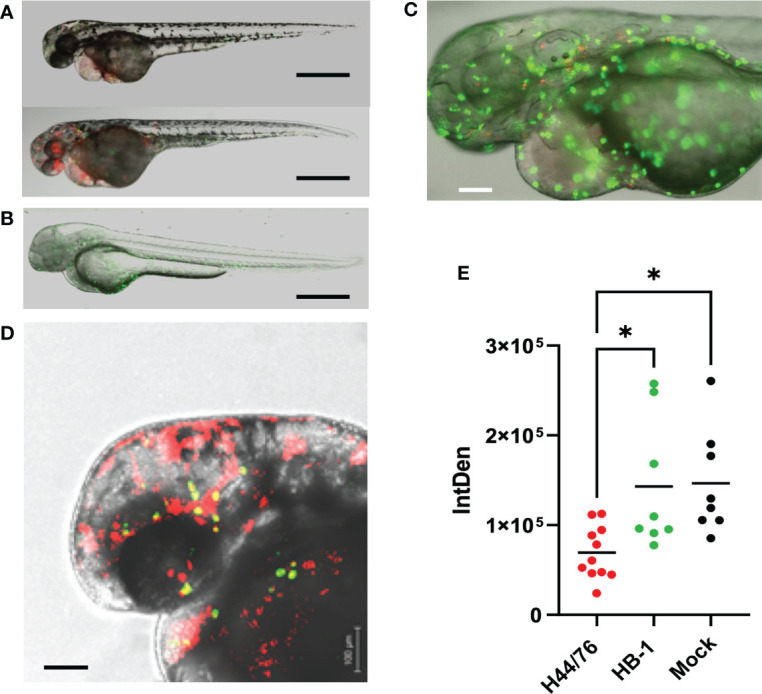
Fluorescence microscopy of H44/76 meningococci expressing mCherry (red fluorescence) and neutrophils expressing GFP (green fluorescence) in zebrafish embryos. **(A)**. H44/76 infected embryos at 24 hpi, Bar represents 100 μm. **(B)**. HB-1 infected embryo at 24 hpi. **(C)**. Detail of H44/76 infected embryo showing green fluorescent neutrophils and red fluorescent meningococci. **(D)** Detail of H44/76 infected embryo showing green fluorescent neutrophils and red fluorescent meningococci in the embryo head. Yellow color shows overlap between meningococci and neutrophils.Scale bars represent 100 μm. **(E)** Number of neutrophils in zebrafish embryos 24hpi of *N. meningitidis* H44/76 serogroup B (1400 cfu) or non-encapsulated HB-1 (1600 cfu) and mock-infected; 11 embryos infected with H44/76, two groups of 8 embryos infected with HB-1 or mock infected, respectively. IntDen = Integrated density of the green fluorescence signal in the area of interest. * P = < 0.05.

To analyze neutrophil behavior *in vivo* upon meningococcal infection, transgenic zebrafish embryos (28 hpf) harboring green neutrophils (mpo:eGFP), were inoculated with red fluorescent H44/76_B (1400 cfu) or HB-1 (1600 cfu). Neutrophils were quantified by measuring the amount of green fluorescence **(** **Figures 4A–D** **)**. At 24 hpi, the number of neutrophils was 80% lower in H44/76_B infected embryos than in mock-infected embryos (P<0.0001). In contrast, embryos infected with non-encapsulated HB-1 meningococci showed similar neutrophil numbers compared to mock-infected embryos (**Figure 4E** **).** Together these results show that the meningococcal polysaccharide capsule is essential to multiplication of meningococci in fish embryos and fish embryo mortality. Meningococcal multiplication, depletion of neutrophils and mortality in zebrafish embryos appears to be correlated.

### Structure of meningococcal capsule affects zebrafish embryo mortality after infection

**Figure 5:**
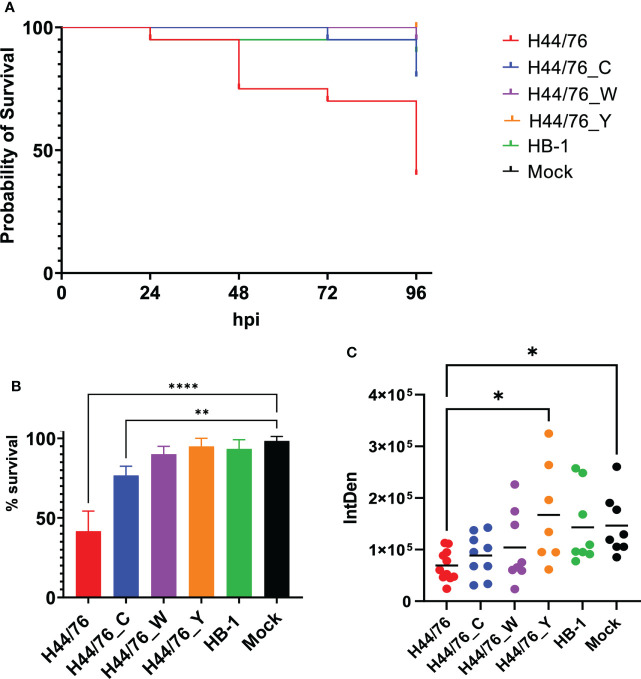
**(A)** Survival curve of zebrafish embryos infected with H44/76 expressing serogroup B capsule (1560 cfu), no capsule (HB-1) (3600 cfu), serogroup C capsule (H44/76_C) (1200 cfu), serogroup W capsule (H44/76_W) (3600 cfu) or serogroup Y capsule (H44/76_Y) (4800 cfu) all expressing mCherry 20 embryos in each group. **(B)** Survival of zebrafish embryos infected with H44/76 expressing serogroup B capsule (2700 ± 1820 cfu), no capsule (HB-1) (1960 ± 1420 cfu), serogroup C capsule (H44/76_C) (2160 ± 1660 cfu), serogroup W capsule (H44/76_W) (2760 ± 840 cfu) or serogroup Y capsule (H44/76_Y) (2720 ± 1817 cfu) scored at 96hpi. Three independent experiments, 20 embryos in each group. **(C)** Quantification of GFP-expressing neutrophils in zebrafish embryos 24 hpi with *N. meningitidis* H44/76_B (1400 cfu), HB-1 (1600 cfu), H44/76_C (1400 cfu), H44/76_W (4000 cfu), H44/76_Y (3000 cfu) or PBS (Mock); 11, 8, 9, 8, 7 and 8 embryos per group, respectively. IntDen = Integrated density of the green fluorescence signal in the area of interest. * P = < 0.05, ** P = < 0.01, **** P = < 0.0001.

Studies with human complement indicate the relevance of the meningococcal capsule polysaccharide structure. However, to the best of our knowledge, studies using true isogenic variants expressing different capsule in identical genetic background are lacking. We constructed isogenic variants of *N. meningitidis* H44/76 by switching their expression of serogroup B capsule to serogroup C (H44/76_C), serogroup W (H44/76_W) or serogroup Y capsule (H44/76_Y). All variants grew equally well in TSB at 28°C (**Supplemental Figure 3**). In addition, all serogroup variants expressed their capsule at 28°C as confirmed by serogrouping, as assessed by Ouchterlony gel diffusion, a classical immunoprecipitation assay (Slaterus, 1961) (**Supplemental Figure 4**). The survival at 96 hpi of embryos infected with H44/76_B (2700 cfu), H44/76_C (2160 cfu), H44/76_W (2760 cfu), H44/76_Y (2720 cfu) or the non-encapsulated HB-1 (1960 cfu) was 42 ± 13%, 77 ± 6%, 90 ± 5%, 95 ± 5% and 93 ± 6%, respectively, while 98 ± 3% of mock-infected embryos survived (**Figures 5A, B** **).** The proportion of embryos that survived after infection with H44/76_B or H44/76_C differed significantly from that of mock-infected embryos (P<0.01) (**Figure 5B** **).**

At 24 hpi, the number of neutrophils was 53% lower in embryos infected with H44/76_B than in mock-infected embryos (P<0.05) (**Figure 5C**). In addition, the number of neutrophils was significantly lower in H44/76_B infected embryos compared to H44/76_Y or HB_1 (**Figure 5B**) (P<0.05). In embryos infected with HB-1, H44/76_C, H44/76_W or H44/76_Y neutrophil numbers were not significantly different from that in mock-infected embryos (**Figure 5C** **).**

**Figure 6:**
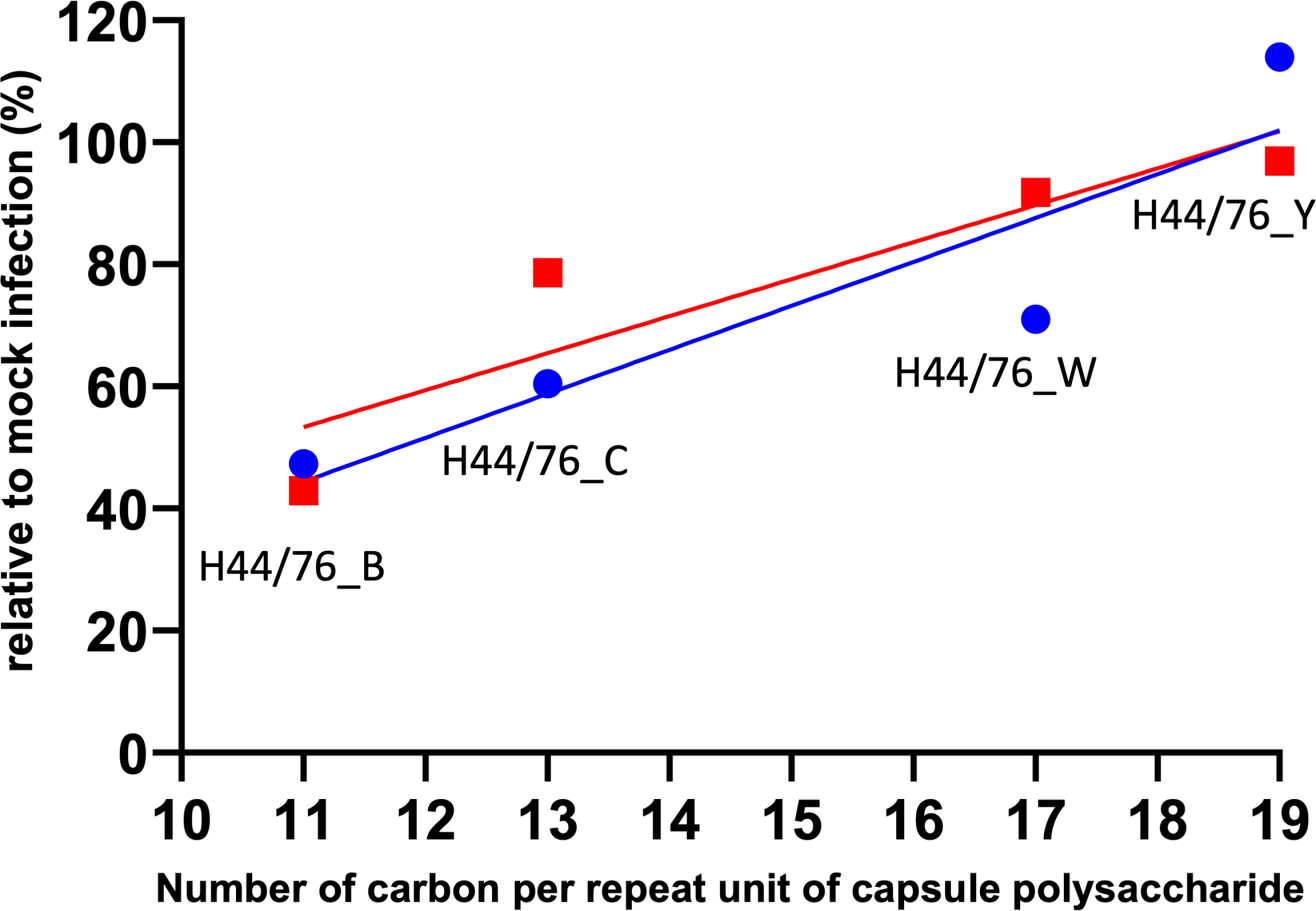
Relation between metabolic cost and meningococcal virulence in zebrafish embryos. Lines represent linear regression. Red: Zebrafish embryo survival after 96 hours post infection with either of the four isogenic capsule variants is the ratio between the number of embryos survived after meningococcal infection and after mock infection (r=0.9090). Blue: Integrated density of the green fluorescence signal as a measure of the number of neutrophils in the area of interest after 24 hours post infection with either of the four isogenic capsule variants divided by that of mock infection; neutrophil count relative to mock-infected zebrafish embryos (r=0.9097).

The number of carbons per repeat unit of capsular polysaccharide is indicative of the metabolic cost incurred during biosynthesis of the capsule (Weinberger et al., 2009). By examining published polysaccharide structures, we determined the number of carbons per one polysaccharide repeat unit of the serogroup B, C, W and Y polysaccharide. Of note, the polysialic acid of the serogroup B capsule is not O-acetyl decorated since the gene encoding the O-acetyltransferase is lacking in the cps locus of serogroup B isolates. The serogroup specific region in the cps locus of the serogroup C and Y isolate used as donor to generate the isogenic capsule variants does contain the gene encoding the O-acetyltransferase, while that of the serogroup W donor isolate lacks the O-acetyltransferase. This resulted in different numbers of carbons per polysaccharide repeat unit, with serogroup B the lowest and serogroup Y the highest number of carbons per repeat unit (**Figure 6** **).** Embryo killing capacity as well as embryo neutrophil depletion after infection of the isogenic capsule variants were clearly correlated with the number of carbons incorporated in their respective capsule polysaccharide (**Figure 6** **).** Together, these data suggest that the structure of the capsular polysaccharide is predicts meningococcal virulence in zebrafish embryos and is associated with the metabolic cost of the biosynthesis of the meningococcal capsule.

## Discussion

Here, we describe for the first time the establishment of meningococcal infection in the zebrafish embryo infection model system. We showed that encapsulated meningococci can replicate in zebrafish embryos, causing a lethal systemic infection. The infected zebrafish embryos often showed pericardial edema, which is also observed when the embryos are injected with lipopolysaccharide (LPS) (Hsu et al., 2018). These observations suggest a correlation to pulmonary edema which has been reported in patients with acute endotoxemia and in patients with severe meningococcal septicemia (Finley et al., 1975; Eisenhut et al., 2006). Infection with non-encapsulated meningococci was not lethal and the fish embryos were able to clear the infection. This fits with the observation that non-encapsulated meningococci rarely cause invasive meningococcal disease and when this occurs, the patients have comorbidities such as complement deficiencies. The meningococcal capsule protects against killing by complement, while non-encapsulated meningococci are extremely sensitive to it (Mikucki et al., 2022). In this respect, the zebrafish embryo infection model reflects the human requirements for meningococcal infection.

A wide variety of pathogenic bacteria has been studied using zebrafish models, offering a unique view of the cellular response to infection *in vivo* (Torraca and Mostowy, 2018). In our study, large inoculates are required to establish infection and host killing. Zebrafish embryos were resistant to low doses of encapsulated meningococci and cleared the infection (**Figure 3** **)**. Similar findings were reported in *Pseudomonas aeroginosa* zebrafish embryo infection studies (Brannon et al., 2009) as well as in *Shigella flexneri* zebrafish larvae infection studies (Mostowy et al., 2013). In contrast, using *Mycobacterium marinum* or *Salmonella arizonae*, an inoculum of <10 CFU was already sufficient for killing in this model (Davis et al., 2002; van der Sar et al., 2003). In addition, non-pathogenic laboratory strains of bacteria, such as *Escherichia coli* K12 or *Bacillus subtilis*, are rapidly eradicated by zebrafish embryos (Herbomel et al., 1999). While infections by non-encapsulated bacteria are rapidly cleared due to complement activity, infection with bacteria resistant to complement are controlled by phagocytic cells. Successful infection in zebrafish embryos most likely then depends on the number of bacteria injected, the multiplication rate of the bacteria and the number of phagocytic cells. With a low multiplication rate in zebrafish embryos, which are kept at 28°C, and low number of bacteria injected, bacteria are outcompeted by the phagocytic cells, whereas high multiplication rates and/or high inoculates results in phagocyte depletion and bacterial overgrowth, resulting in embryo death (Mostowy et al., 2013). Neutrophil depletion has been observed after infection of zebrafish larvae with *Salmonella enterica* serovar Typhimurium (Hall et al., 2012), S*taphylococcus aureus* (Prajsnar et al., 2008) and *S. flexneri* (Mostowy et al., 2013) infections. In zebrafish embryos infected by *S. flexneri*, the numbers of phagocytic cells dramatically decreased in embryos unable to control the bacterial proliferation, and leukocyte depletion was associated with bacteremia preceding death of the embryos (Mostowy et al., 2013). Here, we describe similar findings with zebrafish embryos infected with meningococci. We observed meningococcal propagation and neutrophil depletion when embryos were infected with encapsulated meningococci. This fits with previous observations that invasive meningococcal disease is characterized by striking abnormalities in absolute neutrophil count, immature neutrophil count and immature-to-total neutrophil ratio (Demissie et al., 2013). In addition, neutropenia was associated with poor prognosis (Demissie et al., 2013). Together, these data are indicative of an effective defense mechanism against systemic infection by meningococci in zebrafish embryos that depends solely on innate immunity since adaptive immunity has not yet developed in these embryos.

In the absence of the adaptive immune system, efficient phagocytosis of meningococci is driven by opsonization with complement and requires complement activation (Krüger et al., 2018). The meningococcal capsular polysaccharide hampers phagocytosis by increasing the negative charge of the bacterial surface (Jarvis and Vedros, 1987). In addition, capsular polysaccharides from different serogroups differ in their activation of the alternative complement pathway (Ram et al., 2011). Together, the structure of the capsular polysaccharide may affect the efficiency of phagocytosis. The half-life of neutrophils is only several hours and although meningococci have mechanism to postpone apoptosis, reduction of the number of neutrophils most likely occurs after phagocytosis of meningococci followed by apoptosis (Criss and Seifert, 2012). Interestingly, the meningococcal capsule has been implicated in intracellular survival (Spinosa et al., 2007). However, there are no public data available suggesting a specific role for the meningococcal capsular polysaccharide structure in intracellular survival in neutrophils. Together, efficacy of phagocytosis, intracellular survival and thus reduction of neutrophil numbers may be affected by the specific structure of the capsular polysaccharide.

We report for the first time the generation of isogenic capsule variants to study the impact of the capsule polysaccharide structure and the host’s immune system. We constructed variants in an serogroup B strain, H44/76, to create isogenic serogroup C, W and Y strains, but did not succeed to generated the serogroup A variant. Possibly, the serogroup A capsule is not compatible with the genetic background of clonal complex 32 (cc32). Interestingly, transformation of a serogroup A isolate into a serogroup B isolate was demonstrated, but the reversed experiment transforming a serogroup B isolate into a serogroup A isolate was not reported (Swartley et al., 1998). This observation is consistent with the notion that in PubMLST 23 clonal complexes were identified among 1836 serogroup/genogroup A isolates with known clonal complex annotation, but none belonged to cc32 (https://pubmlst.org/organisms/neisseria-spp; Jolley et al., 2018).

Our results using isogenic capsule variants of *N. meningitidis* H44/76 showed that the meningococcal capsule polysaccharide structure affects the zebrafish embryos killing capacity as well as neutrophil depletion after infection. This could be explained by differential complement activation and clearance of these capsule variants. As aforementioned, meningococcal isolates with different serogroups vary in their interaction with complement (Ram et al., 2011; Lewis and Ram, 2020). However, in these studies the strains with different serogroups did not have the same genetic background. Weinberger and colleagues demonstrated an association between polysaccharide structure, degree of encapsulation and susceptibility to neutrophil-mediated killing in *Streptococcus pneumoniae* (Weinberger et al., 2009). It was proposed that serotypes that produce metabolically inexpensive polysaccharides, estimated by the number of carbons per simple polysaccharide repeat unit, are more heavily encapsulated, resulting in an increased resistance to surface phagocytosis by human neutrophils *in vitro*  (Weinberger et al., 2009). In addition, Ly and colleagues showed that an epidemiological measure of competitive ability to colonize, mean duration of carriage in young children, and resistance to phagocytosis *in vitro*, correlate negatively with cell surface charge, which was measured as zeta potential (Li et al., 2013). Also, the hierarchy in rank of pneumococcal capsular types in mouse colonization correlated with the metabolic cost of capsule synthesis and pneumococcal cell zeta potential (Trzciński et al., 2015). We did not measure the zeta potential of the different meningococcal isogenic capsule variants. However, upon infection of zebrafish embryos with meningococcal isogenic capsule variants, we observed a correlation between capsular polysaccharide structure, zebrafish killing capacity, neutrophil depletion and number of carbons per polysaccharide repeat unit. Hence, the observed differences between the meningococcal isogenic capsule variants in zebrafish embryo virulence may be simply a matter of differences in metabolic cost instead of differences in molecular interactions with host immune components.

In conclusion, the zebrafish embryo infection model represents a valuable new system for the analysis of *N. meningitidis* infection. The model could be useful to study host-pathogen interaction, in particular the interaction between the innate immune system and meningococci. In addition, interactions between bacteria and host cells, like those around the blood brain-barrier can be imaged at high resolution *in vivo*. Here, we demonstrated that meningococcal virulence in the zebrafish embryo largely depends on the presence of the polysaccharide capsule. The extent of this contribution is determined by their structures and may simply reflect differences in trade-offs between virulence and the metabolic costs of capsule production.

## Data availability statement

The datasets presented in this study can be found in online repositories. The names of the repository/repositories and accession number(s) can be found below: https://pubmlst.org/organisms/neisseria-spp, id:119505; https://pubmlst.org/organisms/neisseria-spp, id:20477; https://pubmlst.org/organisms/neisseria-spp, id:55117; https://pubmlst.org/organisms/neisseria-spp, id:119479; https://pubmlst.org/organisms/neisseria-spp, id:61394; https://pubmlst.org/organisms/neisseria-spp, id:119482; https://pubmlst.org/organisms/neisseria-spp, id:61397; https://pubmlst.org/organisms/neisseria-spp, id:119480.

## Ethics statement

Ethical review and approval was not required for the animal study because Adult zebrafish were handled in compliance with the local animal welfare regulations approved by the local animal welfare committee (DEC) and were maintained according to standard protocols (www.zfin.org). Experiments with zebrafish embryos younger than 5 days post fertilization (5 dpf) were excepts for ethical approval.

## Authors contribution

KS, YP and AE conceived the study. KS and LP performed the experiments and collected the data. KS and AE wrote the first draft of the manuscript. All authors contributed to the article and approved the submitted version.

## Funding

Funding for this investigator-initiated research was provided by a grant from Pfizer: WI242174. The funder was not involved in the study design, collection, analysis, interpretation of data, the writing of this article or the decision to submit it for publication. All authors declare no other competing interests.

## Acknowledgments

We like to thank Dr. Dave Speijer for stimulating discussion, many useful suggestions and critical review of the manuscript.

## Conflict of interest

The authors declare that the research was conducted in the absence of any commercial or financial relationships that could be construed as a potential conflict of interest.

## Publisher’s note

All claims expressed in this article are solely those of the authors and do not necessarily represent those of their affiliated organizations, or those of the publisher, the editors and the reviewers. Any product that may be evaluated in this article, or claim that may be made by its manufacturer, is not guaranteed or endorsed by the publisher.
