# Supplementary material for: Meningococcal virulence in zebrafish embryos depends on capsule polysaccharide structure
Source: Front Cell Infect Microbiol. 2022 Sep 23;12:1020201. doi: 10.3389/fcimb.2022.1020201 (PMC9538531; doi:10.3389/fcimb.2022.1020201)
Supplement: Supplementary file 1 [file DataSheet_1.pdf]

Supplemental data

Detailed protocol to generate capsule variants of H44/76

Serogroup B_W gen fusion was constructed according the scheme presented in Supplementary Figure 1. All single fragments (primers presented in Supplemental Table 1 were amplified by using the Phusion Hot Start II High-Fidelity DNA Polymerase kit *N. meningitidis* serogroup W, C and Y DNA or pAErmC' plasmid DNA were used as input. The cycling parameters used are shown in Supplemental Table 2A. PCR products were individually purified using the GeneJET PCR purification kit (Thermo Fisher Scientific®) and the purified products were analyzed by electrophoresis. To fuse the 4 single DNA fragments together a PCR-based gene fusion step (a PCR reaction without primers) was used according to Shevchuc *et al.*, 2004.

The product mix is used in the first fusion step of 13 cycles. The cycling parameters of all fusion steps are shown in Supplemental Table 2B. The fused product was then again purified using the GeneJET PCR purification kit (Thermo Fisher Scientific®) and eluted in 30 µl. An aliquot of 10 µl of the purified fused product was used in the final step using the nested primers. The cycling parameters are shown in Supplementary Table 1B.

The fused product was transformed to H44/76 by natural transformation

Supplemental Table 1. Strains and Primers used in PCR based gene fusion

Strain (id in PubMLST)	primer		Annealing site
HB-1 (119505)			
H44/76 (20477)			
donor Nm W 2170958 (55117)			
transformant H44/76_W (119479)	LPNMWFK	GTTTGATTTGTTTGGCGAACTCGACGG	Forward first fragment
	LPNMWRK	GTCTTTTGCGCATTCAGACGGCGATCGATCAAGCCGAATTCATGCAAACCGCAAACAAAAAAACCATCC	Reverse first fragment
	LPERYCASSF	CGCCGGTAACGAGGATGGTTTTTTTGTTTGCGGTTTGCATGAATTCGGCTTGATCGATCGCCGTCTGAATG	Forward second fragment
	LPERYCASSR	GCGTTGGGTCAGCCGCAACCCCAACGGATATGGGGATTAAGAATTCGGCTTCATGCTCTGACGTCTAAATG	Reverse second fragment
	LPNMWFL	GCATTAGTGCATTTAGACGTCAGAGCATGAAGCCGAATTCTTAATCCCCATATCCGTTGGGGTTGCGGCT	Forward third fragment
	LPNMWNRL1	GCTAAATTTCATGGTATTTCCAAAGAGATGTTTTATCTGCATCTCAAAGAAACGGAGTTTAGGTTTAACC	Reverse third fragment
	LPNMWNFL2	TCATGCCGGTGGTTAAACCTAAACTCCGTTTCTTTGAGATGCAGATAAAACATCTCTTTGGAAATACCATG	Forward fourth fragment
	LPNMWRL	CTTTAATGGCGGAGATACTCGCACCAGCAC	Reverse fourth fragment
donor Nm Y 2170130 (61394)			
transformant H44/76_Y (119482)	KSNMYF	GTTTGAGCTGTTTGGCGAACTCG	Forward first fragment
	LPNMWRK	GTCTTTTGCGCATTCAGACGGCGATCGATCAAGCCGAATTCATGCAAACCGCAAACAAAAAAACCATCC	Reverse first fragment
	KSeryF2	GAATTCGGCTTGATCGATCGCCGTCTGAATGCGCAAAAGAC	Forward second fragment
	KSeryR2	GAATTCGGCTTCATGCTCTGACGTCTAAATG	Reverse second fragment
	LPNMWFL	GCATTAGTGCATTTAGACGTCAGAGCATGAAGCCGAATTCTTAATCCCCATATCCGTTGGGGTTGCGGCT	Forward third fragment
	LPNMWRL1	GCTAAATTTCATGGTATTTCCAAAGAGATGTTTTATCTGCATCTCAAAGAAACGGAGTTTAGGTTTAACC	Reverse third fragment
	LPNMWNFL2	CATGCCGGTGGTTAAACCTAAACTCCGTTTCTTTGAGATGCAGATAAAACATCTCTTTGGAAATACCATG	Forward fourth fragment
	LPNMWRL	CTTTAATGGCGGAGATACTCGCACCAGCAC	Reverse fourth fragment
donor Nm C 2170154_A (61397)			
transformant H44/76_C (119480)	LPNMWFK	GTTTGATTTGTTTGGCGAACTCGACGG	Forward first fragment
	KSNMCR	GTCTTTTGCGCATTCAGACGGCGATCGATCAAGCCGAATTCATGAGAAAAATCCTTGTTACCGGCGGTG	Reverse first fragment
	KSeryF2	GAATTCGGCTTGATCGATCGCCGTCTGAATGCGCAAAAGAC	Forward second fragment
	KSeryR2	GAATTCGGCTTCATGCTCTGACGTCTAAATG	Reverse second fragment
	KSBMCF2	GCATTAGTGCATTTAGACGTCAGAGCATGAAGCCGAATTCAAATTTCAGAGGATAGCCAAAAAATATAAAC	Forward third fragment
	KSNMCR2	CAATAAATATATTTAAGCTGGAGTCTTATTCATCATGTCAATCAATACGTTTGAAACCTTTATTTACGAATTATACC	Reverse third fragment
	KSNMCF3	CTTTAATGGCGGAGATACTCGCACCAGCAC	Forward fourth fragment
	LPNMWRL	GGTATAATTCGTAAATAAAGGTTTCAAACGTATTGATTGACATGATGAATAAGACTCCAGCTTAAATATATTTATTG	Reverse fourth fragment

Supplemental Table 2. Cycling parameters PCR experiments

(A) Cycling parameters used in normal PCR experiments with primers. (**B)** Cycling parameters used during gene fusion with a PCR, no primers were used in this step.

(A)

	First fragment	Second fragment	Third fragment	Fourth fragment	Complete fragment
Step 1 1 cycle	98°C 2 min	98°C 2 min	98°C 2 min	98°C 2 min	98°C 2 min
Step 2 30 cycles	98°C 30 sec	98°C 30 sec	98°C 30 sec	98°C 30 sec	98°C 30 sec
Step 3	60°C 30 sec	60°C 30 sec	60°C 30 sec	60°C 30 sec	60°C 30 sec
Step 4	72°C 1,5 min	72°C 1 min	72°C 2 min	72°C 3 min	72°C 6 min
Step 5 1 cycle	72°C 5 min	72°C 5 min	72°C 5 min	72°C 5 min	72°C 5 min
Step 6	4°C hold	4°C hold	4°C hold	4°C hold	4°C hold

(B)

	Fragment 1+2+3+4			All 4 fragments
Step 1 1 cycle	98°C 2 min		Step 1: 1 cycle	98°C 2 min
Step 2 13 cycles	98°C 30 sec		Step 2: 20 cycles	98°C 30 sec
Step 3 13 cycles	55°C 30 sec		Step 3: 20 cycles	60°C 30 sec
Step 4 13 cycles	72°C 2,5 min		Step 4: 20 cycle	72°C 6 min
Step 5 1 cycle	72°C 5 min		Step 5: 1 cycle	72°C 5 min
Step 6	4°C hold		Step 6	4°C hold

Supplemental Figure 1.

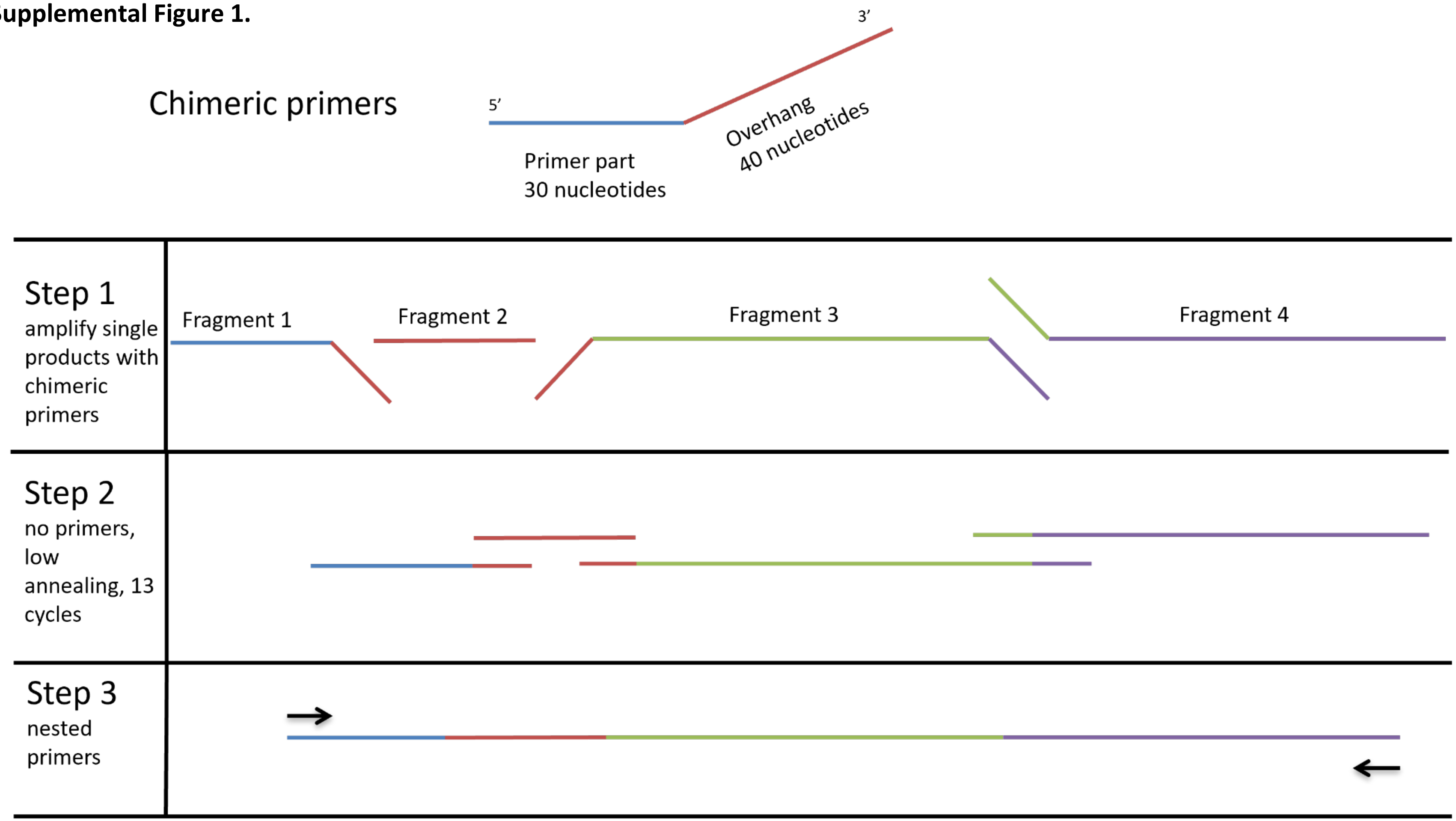

PCR based gene fusion for the generation of the complete DNA construct using 2 fusion steps.

Supplemental Figure 2A.

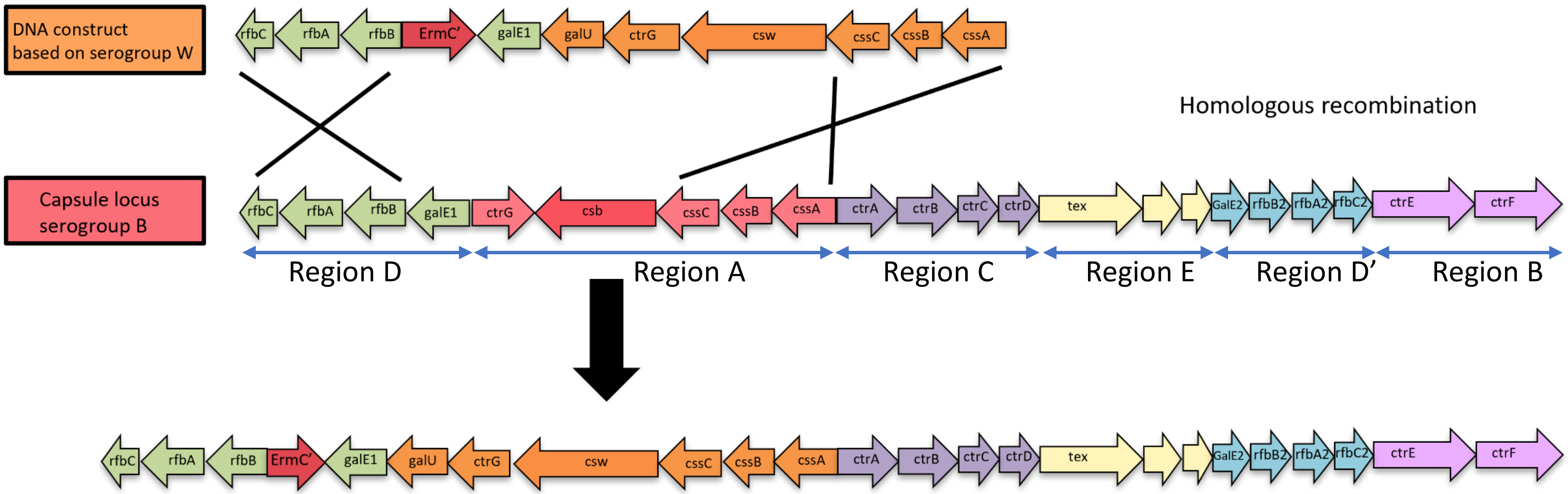

Construction of *N. meningitidis* H44/76 expressing serogroup W. Annotation of the different region is according to Harrison *et al*., 2013 *Emerg. Infect. Dis*. 19, 566-73. doi: 10.1016/j.stem.2012.01.007.

Supplemental Figure 2B.

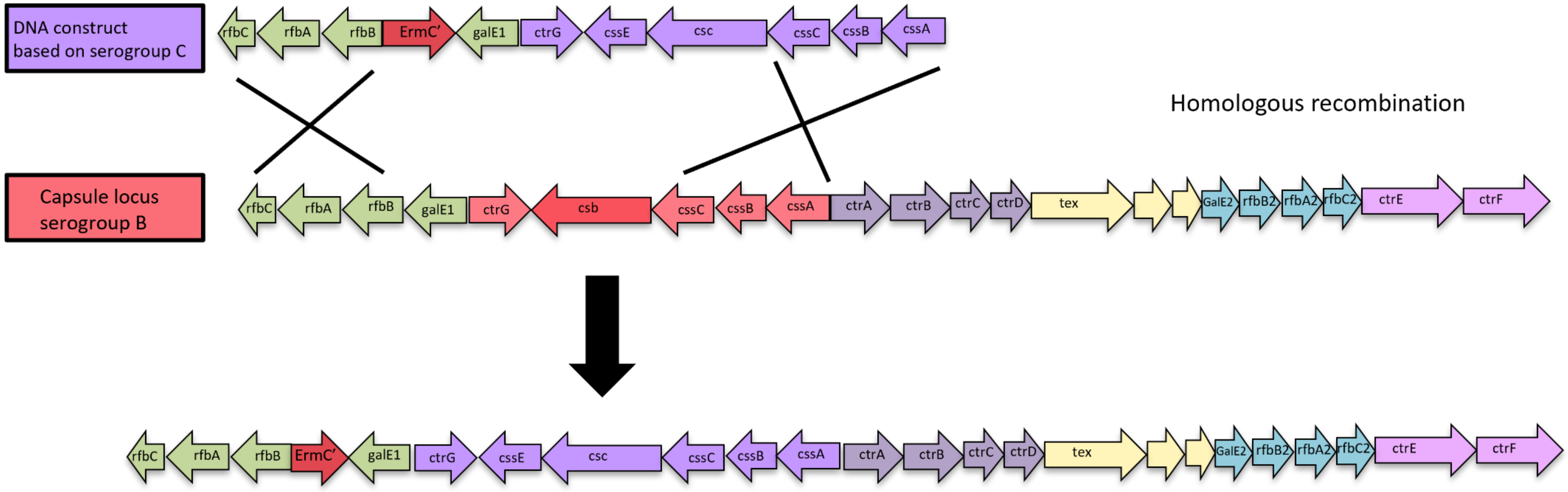

Construction of *N. meningitidis* H44/76 expressing serogroup C

Supplemental Figure 2C.

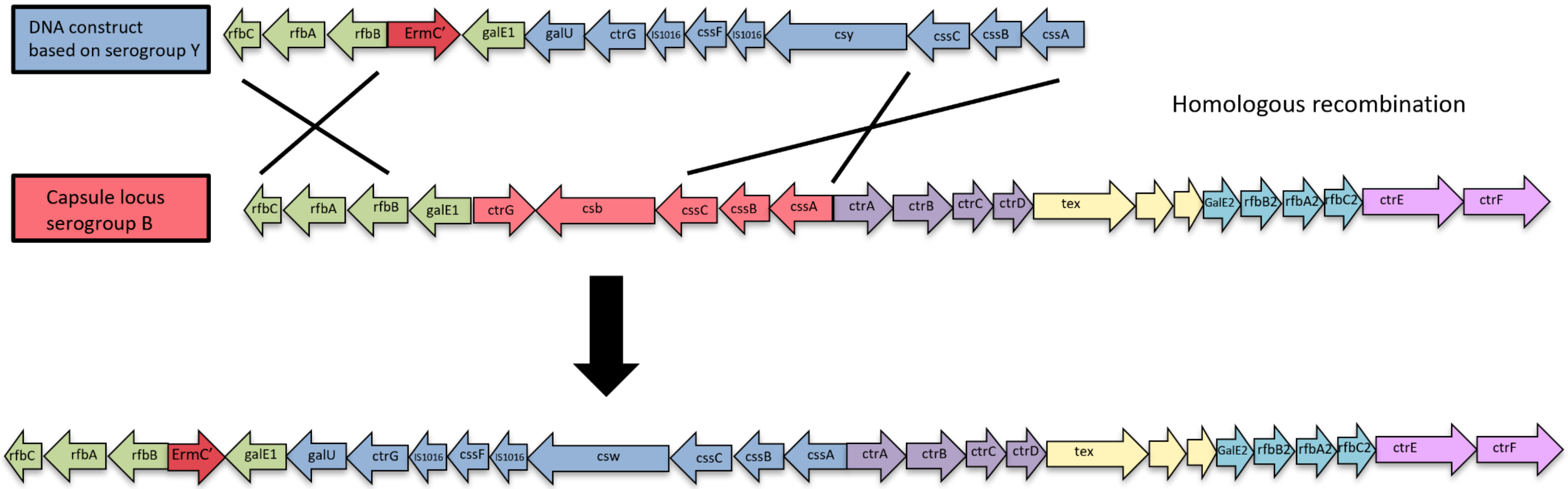

Construction of *N. meningitidis* H44/76 expressing serogroup Y

Supplemental Figure 3.

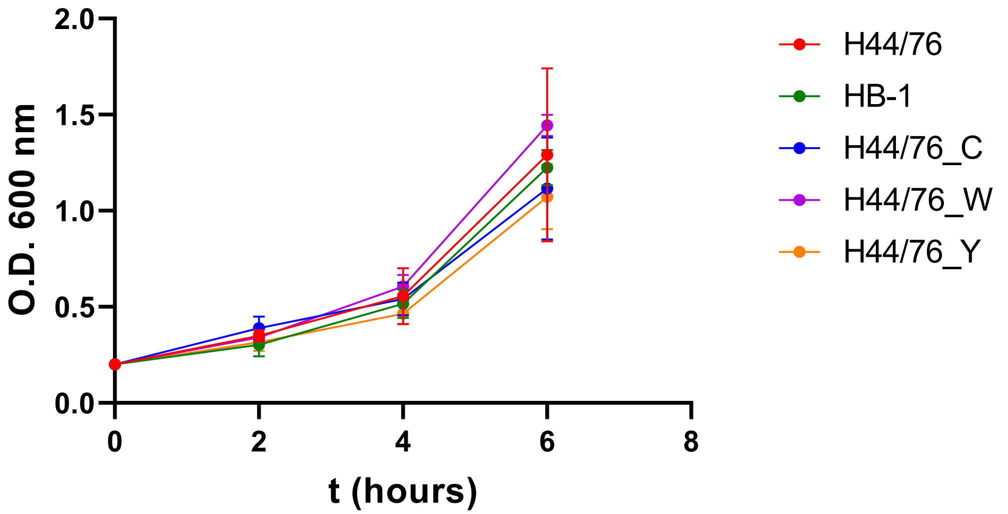

Growth of H44/76 serogroup B, non-encapsulated HB-1 and the isogenic capsule variants of H44/76 in Tryptic Soy Broth at 28°C. Data of three biological replicates each plated in triplicate on GC agar plates. Error bars represent standard deviations of the mean of the three biological replicates.

Supplemental Figure 4.

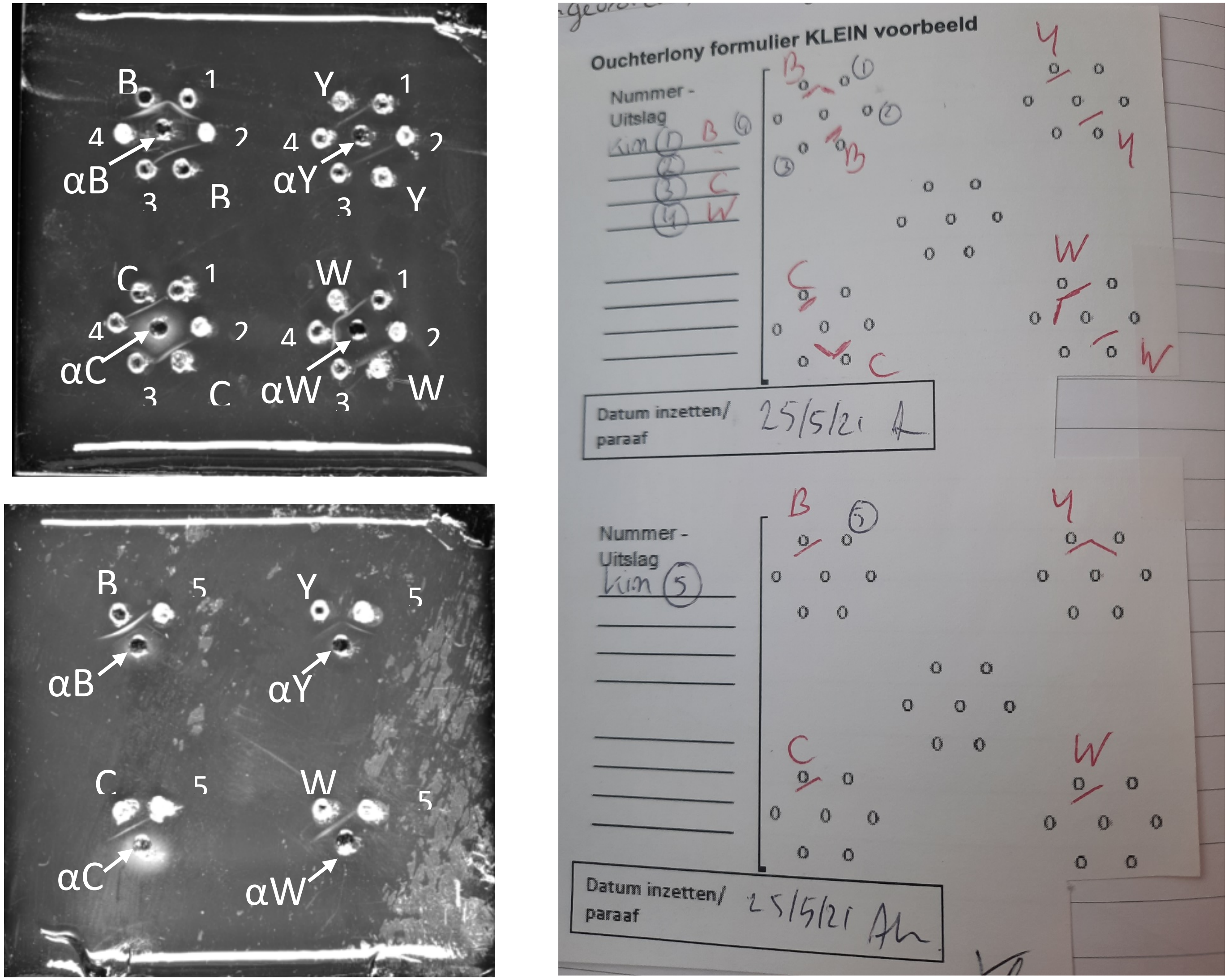

Ouchterlony gel diffusion of the meningococcal isogenic capsule variants. 1. H44/76; 2. HB-1; 3. H44/76_C; 4. H44/76_W; 5. H44/76_Y. B, C, W and Y represent control strains for serogroup B, C, W and Y respectively. αB, αW and αY indicate the wells in which antiserum is supplied against serogroup B, serogroup C, serogroup W and serogroup Y, respectively.
